# Supplementary material for: The Influence of Intensive Nutritional Education on the Iron Status in Infants—Randomised Controlled Study
Source: Nutrients. 2025 Sep 29;17(19):3103. doi: 10.3390/nu17193103 (PMC12525554; doi:10.3390/nu17193103)
Supplement: Supplementary file 1 [file nutrients-17-03103-s001.zip › nutrients-3836723-supplementary.pdf]

**Suplemmentary data**

**Table S1.** Comparison of dietary intake between the study group and the control group at the baseline of the trial.

| Dietary intake (% RDA) | Study group (n=52) **Baseline** | | Control group (n=52) **Baseline** | | *p* |
|---|---|---|---|---|---|
| | Median (Q1–Q3) | Mean ± SD (95% CI) | Median (Q1–Q3) | Mean ± SD (95% CI) | |
| Iron | 49.3 (15.2–110.3) | 70.0 ± 69.5 (37.7–104.4) | 52.9 (15.4–104.4) | 63.5 ± 47.9 (32.3–93.8) | 0.9823 [1] |
| Proteins | 145.1 (57.5–242.7) | 164.2 ± 127.2 (105.0–232.9) | 179.5 (97.3–399.5) | 225.0 ± 195.5 (147.6–313.6) | 0.2797 [1] |
| Vitamin C | 70.0 (47.5–104.2) | 96.8 ± 77.1 (50.2–143.4) | 65.5 (46.6–140.9) | 93.6 ± 69.5 (49.3–137.78) | 0.8918 [1] |

RDA – Recommended Dietary Allowance according to Polish nutritional standard

[1] Mann–Whitney U test; [2] Student’s t-test
